# Plants used in medicines and foods in osteoporosis: mechanistic insights into bone-remodeling homeostasis and clinical evidence

**DOI:** 10.3389/fphar.2026.1839782

**Published:** 2026-06-01

**Authors:** Mingjun Wang, Huilin Tu, Jun Guo

**Affiliations:** Department of Basic Innovation Research, Beijing Hospital, National Center for Gerontology, National Clinical Research Center for Gerontology, The Key Laboratory of Geriatrics of NHC, Beijing Key Laboratory of Aging Mechanism and Intervention Research on Aging-Related Diseases, Institute of Geriatric Medicine, Chinese Academy of Medical Sciences, Beijing, China

**Keywords:** osteoporosis, plants used in medicines and foods, bone remodeling, bone resorption, osteogenesis

## Abstract

Osteoporosis (OP) arises from the progressive uncoupling of bone remodeling, in which osteoclastic resorption exceeds osteoblastic formation under the combined influence of estrogen deficiency, nutrient insufficiency, oxidative stress, chronic inflammation, and age-related stem-cell decline. Although current antiresorptive and anabolic drugs improve bone mineral density (BMD), their long-term use is constrained by adverse effects, limited impact on bone microarchitecture, and suboptimal patient adherence. Plants used in medicines and foods (PMF), characterized by their dietary origin, multi-metabolite composition, and favorable safety profile, have emerged as promising candidates for sustained OP management. Accumulating evidence indicates that PMF metabolites, including flavonoids, polysaccharides, glycosides, and coumarins, exert coordinated osteoprotective effects by enhancing osteogenic pathways (Wnt/β-catenin, BMP/SMAD), suppressing osteoclastogenic signaling (RANKL/NFATc1/NF-κB), modulating endocrine axes, reshaping the gut-bone and immune microenvironment, and improving aging-related bone marrow-derived mesenchymal stem cell (BMSC) dysfunction. This review synthesizes current mechanistic and clinical evidence supporting PMF-based interventions and highlights critical challenges and the need for large-scale randomized controlled trials (RCTs). These advances will be essential for translating PMF into evidence-based, globally applicable strategies for OP prevention and adjunctive therapy.

## 1 Introduction

Osteoporosis (OP) is a progressive skeletal disorder characterized by reduced bone mass, microarchitectural deterioration, and heightened fracture risk. As a major chronic disease associated with population aging, OP affects nearly one-third of postmenopausal women and one-fifth of men over 50 years old worldwide and imposes a substantial global health and economic burden (Jain, 2024; Zhou et al., 2025). The disease develops insidiously and often remains asymptomatic until fragility fractures occur, leading to chronic pain, disability, loss of independence, and increased mortality (González et al., 2009). At the biological level, OP reflects a fundamental disruption of bone remodeling dynamics, specifically, the uncoupling between osteoclast-mediated resorption and osteoblast-driven formation. This dysregulation is driven and amplified by a convergence of factors, including endocrine changes (estrogen withdrawal), senescence-associated inflammation and oxidative stress, gut microbiota dysbiosis, and age-related impairment of bone marrow-derived mesenchymal stem cell (BMSC) function (Gao, Patil, et al., 2021; Heikkinen et al., 2019; Kaufman et al., 2001; Khosla and Monroe, 2018).

Despite the availability of several pharmacologic agents targeting bone resorption (e.g., bisphosphonates, denosumab) or bone formation (e.g., teriparatide, romosozumab), major limitations persist. Long-term adherence is poor, time-limited use restricts anabolic therapy, and antiresorptives may elicit rare but serious adverse events, including osteonecrosis of the jaw (ONJ) and atypical femoral fractures (AFF) (Diab and Watts, 2013; Papaioannou et al., 2007). Moreover, these drugs primarily act through relatively focused mechanisms and therefore inadequately address the multifactorial and systemic nature of bone deterioration. These constraints underscore an urgent need for safer, multi-target, and sustainable therapeutic strategies, ideally suited for lifelong management, especially for the increasing population requiring extended prevention and maintenance therapy.

In parallel with the search for safer and more sustainable long-term strategies, a growing body of research has explored the role of natural substances in the prevention and management of OP (Perrone et al., 2025). Various diet-derived and plant-based materials, such as medicinal plants, functional foods, and traditional herbal formulations, have been reported to exert beneficial effects on bone health in both experimental and clinical settings. These natural interventions are generally characterized by multi-target and system-level regulatory properties (Heinrich et al., 2022), including the promotion of bone formation, suppression of bone resorption, and modulation of systemic factors such as inflammation, oxidative stress, and endocrine balance. Importantly, their long history of dietary or traditional use suggests a favorable safety profile and suitability for long-term intervention, which is particularly relevant for chronic conditions such as OP.

Within this broader landscape of natural substances, plants used in medicines and foods (PMF), a uniquely integrative framework in traditional Chinese medicine (TCM) that classifies certain natural substances as both dietary metabolites and therapeutic agents, represents a unique and culturally rooted category. Many PMF substances, including *Lycium barbarum* Lam. (Solanaceae), *Pueraria lobata* (Willd.) Ohwi (Fabaceae), *Angelica sinensis* (Oliv.) Diels (Apiaceae), and *Eucommia ulmoides* Oliv. (Eucommiaceae) have historically been used to “strengthen bones and tendons,” and modern research has begun to substantiate these claims. Bioactive metabolites such as flavonoids, polysaccharides, glycosides, and coumarins demonstrate coordinated actions across multiple biological layers: activating Wnt/β-catenin and bone morphogenetic protein/suppressor of mother against decapentaplegic (BMP/SMAD) signaling, suppressing receptor activator of nuclear factor-κB ligand/nuclear factor of activated T cells 1 (RANKL/NFATc1)-mediated osteoclastogenesis, modulating endocrine and nutrient-related pathways (e.g., estrogenic activity and vitamin K-related metabolism), attenuating inflammation and oxidative stress, reshaping the gut-bone axis, and alleviating age-related BMSC functional decline. These multi-level, multi-target properties position PMFs as promising candidates for long-term, low-toxicity, and systemically integrative intervention in OP.

Therefore, this review provides a comprehensive and mechanistically oriented synthesis of current evidence on PMF substances in the regulation of bone remodeling and OP management. We delineate how PMF bioactive metabolites interact with cellular, endocrine, immune-metabolic, and aging-related pathways to restore remodeling balance; summarize preclinical and emerging clinical evidence, together with safety considerations; and outline future research priorities required to advance PMF from traditional practice to evidence-based, globally applicable strategies for OP prevention and adjunctive therapy.

## 2 Methods

### 2.1 Search strategy

**FIGURE 1:**
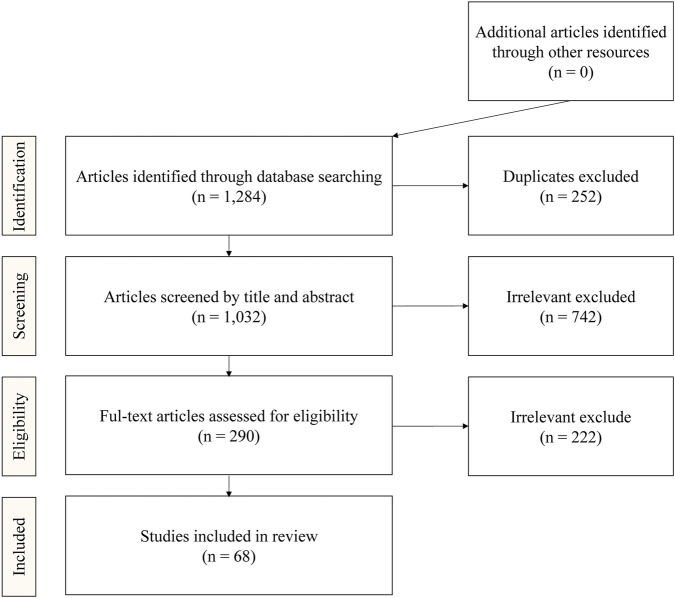
PRISMA flow diagram of the study selection process.

This review is a narrative synthesis rather than a formal systematic review. However, to enhance transparency, reproducibility, and methodological rigor, the literature identification and selection process was conducted using a structured approach informed by the Preferred Reporting Items for Systematic Reviews and Meta-Analyses (PRISMA) guidelines (Figure 1). A systematic search was conducted across multiple electronic databases, including PubMed, Web of Science, Scopus, and Google Scholar.

The search strings were constructed using a combination of MeSH terms and keywords. The primary search terms included: Group 1 (core topic–herbal interventions): “drugs”, “Chinese herbal”, “plant extracts”; Group 2 (specific context–bone loss conditions): “osteoporosis”, “bone loss”; Boolean operators: (Group 1) AND (Group 2).

### 2.2 Inclusion and exclusion criteria

To refine the selection and maintain the quality of the discussed literature, specific inclusion and exclusion criteria were established:

Inclusion criteria: peer-reviewed journal articles and high-quality conference proceedings; studies focused specifically on PMF bioactives in OP.

Exclusion criteria: non–peer-reviewed sources (e.g., blog posts, preprints, theses, or white papers); full text not accessible; not involving PMF substances or relevant bioactive compounds; lacking relevance to bone biology, osteoporosis, or related signaling pathways; providing only peripheral mention of PMF bioactives without mechanistic, pharmacological, or clinical evaluation; insufficient methodological detail to allow critical interpretation (e.g., absence of experimental design description, controls, or outcome measures); duplicate publications or studies with overlapping datasets.

### 2.3 Study selection and data extraction

The selection process followed a two-stage screening approach. First, titles and abstracts were screened for relevance. Second, the full texts of the potentially eligible articles were reviewed to ensure they met the predefined inclusion criteria. Additionally, a “snowballing” manual search was performed by checking the reference lists of the retrieved articles to identify further relevant studies that might have been missed in the initial database search.

A total of 68 articles were finally selected and synthesized in this study.

## 3 Pathogenic mechanisms of OP

**FIGURE 2:**
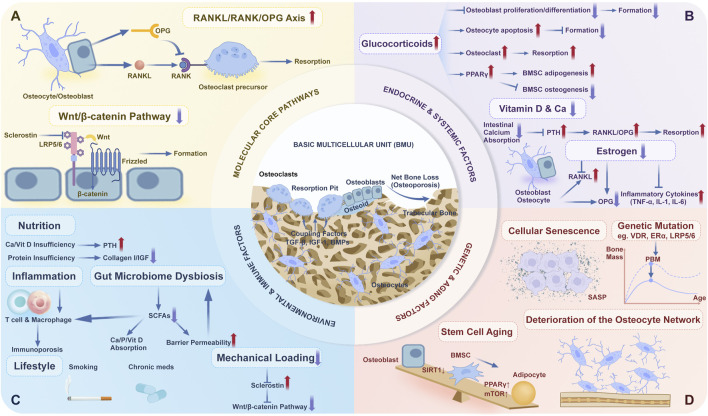
Multilayered pathogenic mechanisms underlying OP ultimately converge on the uncoupling of BMU remodeling.

Physiologically, remodeling is executed within basic multicellular units (BMUs), where osteoclast-mediated resorption and osteoblast-mediated formation proceed in a precisely coordinated sequence to preserve bone mass, microarchitecture, and mechanical competence (Bolamperti et al., 2022; Jilka, 2003). When this coupling is impaired, the net balance shifts toward bone loss and increased fragility. Within the BMU, osteocytes form an extensive lacunar-canalicular network that senses mechanical strain and microdamage (Smit and Burger, 2000). As summarized in Figure 2, OP arises from a four-layer, integrative mechanism framework: (i) core intracellular signaling axes, (ii) hormonal dysregulation, (iii) modifiable environmental and immune drivers, and (iv) genetic architecture and aging-related degeneration, all converging on remodeling uncoupling and net bone loss.

### 3.1 Core molecular pathways driving remodeling imbalance

Bone remodeling is governed by a hierarchical signaling architecture that establishes the local remodeling “set-point.” In OP, disruptions across these control layers converge to uncouple resorption from formation.

At the core of directional control, the RANKL/receptor activator of nuclear factor-κB (RANK)/osteoprotegerin (OPG) axis is the principal determinant of osteoclast commitment. RANKL, produced by osteoblast-lineage cells and osteocytes, binds RANK on osteoclast precursors to activate nuclear factor-κB (NF-κB) and induce NFATc1, promoting osteoclastogenesis; OPG acts as a decoy receptor that neutralizes RANKL (Nelson et al., 2012). An elevated RANKL/OPG ratio is a consistent signature of resorption dominance in estrogen deficiency. Counterbalancing resorption, the Wnt/β-catenin pathway governs osteoblast differentiation (Vlashi et al., 2023). Canonical Wnt ligands stabilize β-catenin through low-density lipoprotein receptor-related protein 5 (LRP5), activating osteogenic transcriptional programs (Bodine and Komm, 2007). Importantly, osteocyte-derived sclerostin inhibits Wnt signaling, and therapeutic sclerostin neutralization restores bone formation, highlighting a key inhibitory checkpoint (Ivanova et al., 2022).

Beyond directional hubs, effective remodeling requires coupling. During osteoclastic digestion, matrix dissolution liberates bioactive molecules, including transforming growth factor-β (TGF-β), insulin-like growth factor-1 (IGF-1), and BMPs, that recruit osteoprogenitors, linking resorption to subsequent osteogenesis (Charles and Aliprantis, 2014). Remodeling outcomes are further refined by epigenetic regulators: miR-218 enhances osteogenesis, whereas miR-148a promotes osteoclastogenesis (Cheng et al., 2013; Hassan et al., 2012). Sirtuin 1 (SIRT1)-dependent histone deacetylation influences osteoblast differentiation (Wu et al., 2023). Collectively, shifts toward increased RANKL signaling and/or suppressed Wnt activity, compounded by impaired coupling and altered sensitivity regulation, provide a coherent molecular basis for remodeling drift and progressive skeletal fragility in OP.

### 3.2 Hormonal dysregulation and systemic bone loss

The endocrine system is a primary upstream determinant of BMU set-points. Among endocrine drivers, estrogen deficiency is the dominant trigger of postmenopausal osteoporosis (PMOP). Estrogen suppresses RANKL while promoting OPG; its loss increases the RANKL/OPG ratio, enhances osteocyte apoptosis, and upregulates sclerostin, collectively shifting remodeling toward a high-turnover, resorption-dominant state (Khosla et al., 2012). Androgens support osteoblast survival through androgen-receptor signaling and aromatization to estrogen; reduced androgen availability weakens bone-forming capacity (Vanderschueren et al., 2004).

A second critical axis is the calcium-vitamin D-parathyroid hormone (PTH) axis. In older adults, vitamin D deficiency reduces intestinal calcium absorption, resulting in secondary hyperparathyroidism. Chronic PTH excess accelerates bone loss (Laird et al., 2010). Notably, this pathologic state contrasts with intermittent PTH exposure, which is anabolic and forms the therapeutic basis for teriparatide (Osagie-Clouard et al., 2017).

A third perturbation, glucocorticoid excess, imposes direct, multi-level suppression of the BMU formation program. Glucocorticoids inhibit osteoblast proliferation and differentiation, induce osteocyte apoptosis, increase RANKL, and skew BMSC fate toward adipogenesis via peroxisome proliferator-activated receptor γ (PPARγ) activation, generating a low-formation, persistent-resorption phenotype (Chen et al., 2023; Liu et al., 2023).

### 3.3 Modifiable environmental and immune contributors to fragility

Nutritional insufficiency is a primary upstream stressor. Inadequate calcium and vitamin D promote a resorptive shift, whereas low protein intake limits type I collagen synthesis and constrains formation capacity (Muñoz-Garach et al., 2020). Mechanical cues provide a second determinant: physiological loading suppresses sclerostin and favors Wnt/β-catenin-dependent osteogenesis, while unloading rapidly accelerates bone loss (Carina et al., 2020; Hejazi et al., 2022; Hughes-Fulford, 2004; Watson et al., 2018).

Immune and inflammatory pathways constitute a major extrinsic route to osteoporotic bone loss. In chronic inflammatory states, activated T cells and macrophages increase immune-derived RANKL and pro-resorptive cytokines (TNF-α, IL-17, IL-6), driving immunoporosis (Srivastava and Sapra, 2022; Wu et al., 2021; Xu et al., 2022). The gut microbiome functions upstream as a systems-level modulator: by shaping vitamin D metabolism, short-chain fatty acid (SCFA) production, and barrier/immune homeostasis, dysbiosis can amplify inflammatory signaling, further biasing BMU set-points toward resorption (Li et al., 2023; Zheng et al., 2025). Additional exposures (e.g., smoking, comorbidities, long-term medications) act largely by intensifying these same intermediary routes.

### 3.4 Genetic architecture and aging-driven remodeling decline

Genetic architecture and biological aging jointly shape the life-course trajectory of skeletal remodeling. Twin and family studies estimate that 50%–85% of interindividual variation in peak bone mass (PBM) is heritable (Chevalley and Rizzoli, 2022). Risk loci cluster in pathways governing BMU function, including vitamin D receptor (VDR), estrogen receptor α (ERα), and Wnt co-receptors LRP5/6; notably, loss-of-function mutations in LRP5 are linked to early-onset osteoporosis (EOOP) (Stürznickel et al., 2020).

Biological aging progressively erodes remodeling efficiency through convergent mechanisms. First, BMSCs undergo quantitative exhaustion and lineage skewing. Reduced SIRT1 activity, heightened PPARγ signaling, and mammalian target of rapamycin (mTOR) activation bias BMSCs toward adipogenesis at the expense of osteogenesis (Bruedigam et al., 2010; Jiang et al., 2020; Sui et al., 2016; Yu et al., 2023). Second, senescent osteoblasts and osteocytes accumulate and acquire a senescence-associated secretory phenotype (SASP) enriched in IL-6 and matrix metalloproteinases (MMPs), disrupting the local remodeling niche (Cheng et al., 2025). Third, deterioration of the osteocyte lacunar-canalicular network compromises mechanosensing and microcrack repair, rendering bone more brittle (Cui et al., 2022; Vashishth et al., 2000).

## 4 Current pharmacological strategies for OP: efficacy and limitations

**FIGURE 3:**
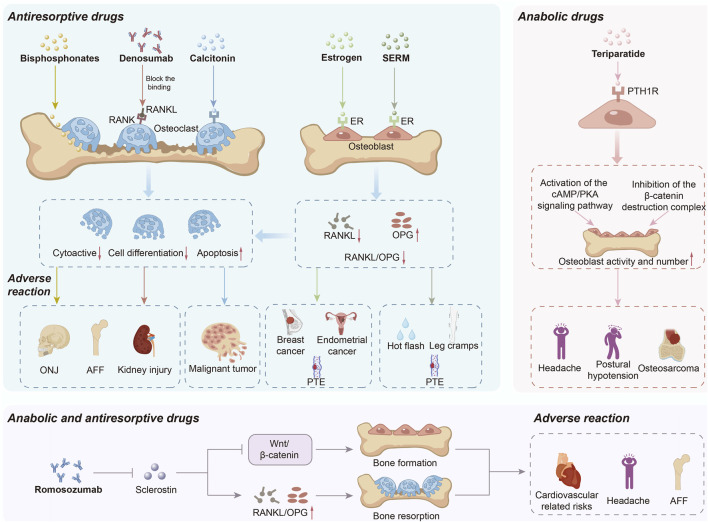
Action mechanisms and adverse reactions of current OP medications.

Current pharmacological management of OP relies primarily on antiresorptive and anabolic agents that reduce fracture risk but are constrained by long-term safety concerns, finite treatment windows, and limited mechanistic breadth (Figure 3).

Among antiresorptives, bisphosphonates (e.g., alendronate, zoledronic acid) are widely used but are associated with rare yet serious adverse events, including ONJ and AFF (Diab and Watts, 2013), as well as gastrointestinal intolerability that impairs adherence. Denosumab, a potent RANKL inhibitor, can lead to a rapid rebound increase in bone turnover upon discontinuation, with a corresponding rise in vertebral fracture risk, mandating vigilant sequential therapy (Anastasilakis et al., 2021). Anabolic agents are limited by treatment duration: teriparatide and abaloparatide are generally restricted to 18–24 months due to concerns over osteosarcoma risk observed in rodent models (Bandeira and Lewiecki, 2022), while romosozumab is typically limited to a single 12-month course to mitigate related-risk, restricting its applicability in chronic disease management (Miller et al., 2021).

Beyond these agent-specific constraints, current therapies share collective limitations: they primarily act through single, focused mechanisms and therefore inadequately address the multifactorial and systemic nature of bone deterioration. These shortcomings underscore an urgent need for safer, multi-target, and sustainable long-term strategies, for which PMF materials, characterized by dietary safety and multi-component pharmacology, represent a promising adjunctive paradigm.

## 5 Theoretical and historical basis of PMF for OP

The concept of PMF is a cornerstone of TCM, referring to natural substances that can function both as foods and as therapeutic agents. By placing dietary intake and health maintenance on a continuum, PMF posits that routine, long-term consumption of selected materials can modulate physiological functions and confer sustained benefits (Chen, 2023). This framing is particularly relevant to OP, a chronic, multifactorial disease that typically requires prolonged prevention and maintenance strategies, yet whose mainstream pharmacotherapies are often limited by finite treatment windows, long-term safety concerns, discontinuation syndromes, and adherence barriers. In this context, PMF materials, characterized by dietary origin, multi-metabolite composition, and a history of long-term use, offer a conceptually attractive adjunctive paradigm aimed at supporting remodeling homeostasis over extended time frames (Chen, 2023).

Classic PMF examples, including *Lycium barbarum, Astragalus membranaceus* Fisch. ex Bunge (Fabaceae), and *Dimocarpus longan* Lour. (Sapindaceae) have long been incorporated into dietary and health-preserving practices, reflecting their dual nutritional and medicinal roles within TCM (Hu et al., 2025). Historically, the philosophical roots of PMF trace back to early medical thought emphasizing prevention and “nourishing” regulation, and the concept gradually matured into a structured dietary-therapy tradition across successive dynasties. Over time, these practices evolved from general principles to increasingly systematized applications, culminating in an integrated culinary-medical framework in which dietary substances were recognized not only for nutrient supplementation but also for their capacity to regulate bodily functions and sustain long-term health.

Crucially, contemporary biomedical research has begun to provide mechanistic plausibility for this traditional framework. Many PMF materials contain bioactive metabolites capable of acting on processes directly implicated in OP pathogenesis, including oxidative stress-related impairment of osteoblast function, inflammation-associated amplification of osteoclastogenic signaling (e.g., RANKL upregulation), and immune-mediated reinforcement of bone resorption (Chen et al., 2024). These findings suggest that PMF substances may operate as multi-target modulators rather than single-pathway drugs, a property that maps onto OP as a systemic disorder driven by convergent endocrine, immune-inflammatory, oxidative, and aging-related pressures that ultimately reset BMU set-points toward net bone loss (Chen, Jia, Han, Zhao, Yang, Xiao, Yang and Yang, 2024).

Building on this convergent historical rationale and emerging mechanistic evidence, PMF offers a plausible long-term intervention paradigm for OP. Fundamentally, OP reflects a sustained imbalance in bone remodeling, characterized by excessive osteoclastic resorption and insufficient osteoblastic formation. Owing to their multi-metabolite and multi-target nature, PMF materials are more likely to act through coordinated modulation of multiple, tightly coupled pathways within the BMU, including osteoclastogenesis, osteogenesis, oxidative stress responses, inflammatory signaling, and immunometabolic regulation, thereby correcting the overall drift of remodeling toward net bone loss. Accordingly, the following sections are structured along a “mechanism-efficacy-translation” evidence chain, moving from pathway-level actions of representative PMF bioactives to *in vivo* effects on bone mass and microarchitecture, and finally to the strength of available clinical evidence (BMD/fracture-related endpoints and safety), thereby clarifying both the opportunities and the current limitations of PMF for OP prevention and adjunctive therapy.

## 6 Coordination of bone remodeling and bone microenvironment by PMF for OP

**FIGURE 4:**
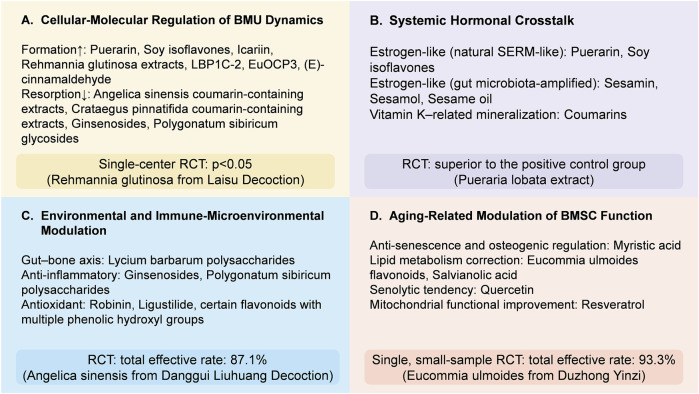
Layered mechanisms underlying the multi-pathway osteoprotective effects and clinical studies of MFH substances.

PMF substances may counter OP via a coordinated, multi-tier regulatory architecture that ultimately resets the remodeling “set-point” within the BMU. Rather than acting on a single dominant target, PMF-derived bioactives can concurrently: (i) rebalance osteoblast-osteoclast output within the BMU, (ii) modulate endocrine signals that bias remodeling toward resorption, (iii) normalize the immunometabolic and environmental milieu shaping BMU activity (e.g., gut-bone axis, inflammation, oxidative stress), and (iv) mitigate aging-associated declines in BMSC function. This layered framework provides a unifying explanation for the multi-pathway osteoprotective effects reported for PMF materials across experimental systems. A consolidated summary of all PMF materials and explicitly discussed bioactives, organized by mechanistic modules, is provided in (Figure 4; Supplementary Table 1, 2).

### 6.1 Cellular-molecular regulation of BMU dynamics

At the execution level, PMF substances act directly within the BMU to restore remodeling balance by promoting osteoblastogenesis, restraining osteoclastogenesis, and improving coupling between resorption and formation. Mechanistically, their effects converge on conserved signaling hubs that govern lineage commitment and functional output, most prominently the Wnt/β-catenin and BMP/SMAD pathways (osteogenic programs) and the RANKL/NF-κB/NFATc1 axis (osteoclastogenic programs).

Multiple PMF-derived flavonoids, including puerarin (*Pueraria lobata*), soy isoflavones (*Glycine max* (L.) Merr. (Fabaceae)), icariin (*Epimedium brevicornu* Maxim. (Berberidaceae)), and *Rehmannia glutinosa* (Gaertn.) Libosch. ex DC. (Orobanchaceae) extracts consistently enhance osteoblast commitment by activating canonical Wnt/β-catenin signaling, increasing nuclear β-catenin and inducing downstream osteogenic transcriptional programs such as runt-related transcription factor 2 (Runx2) and osterix (Osx), thereby promoting osteoblast maturation and extracellular matrix synthesis (Gao, Xiang, et al., 2021; Li et al., 2013; Liu et al., 2019; Mi et al., 2025; Wang et al., 2016; Xu et al., 2021). Complementary pro-osteogenic actions are also observed with polysaccharide fractions, such as LBP1C-2 from *Lycium barbarum* and EuOCP3 from the bark of *Eucommia ulmoides*, which activate BMP-2/SMAD signaling to accelerate osteoblast proliferation, differentiation, and mineral deposition (Song et al., 2024; Sun et al., 2023). Additional phytochemicals, including (E)-cinnamaldehyde from *Cinnamomum cassia* (L.) J.Presl (Lauraceae) further reinforce anabolic signaling by amplifying BMP-2/Runx2 expression (Zhao et al., 2023). Collectively, these studies support a recurring mechanistic theme: PMF metabolites converge on canonical osteogenic nodes to strengthen osteoblast lineage fidelity and functional capacity.

In parallel, PMF substances exhibit antiresorptive actions by suppressing osteoclast differentiation and activation. Coumarin-containing extracts from *Angelica sinensis, Angelica archangelica* L. (Apiaceae), and *Crataegus pinnatifida* Bunge (Rosaceae) can inhibit RANKL-driven NFATc1 induction and downregulate osteoclastic effectors such as cathepsin K (CTSK) and tartrate-resistant acid phosphatase (TRAP) (Abdallah et al., 2019; Kong et al., 2014; Liu et al., 2024; Yan et al., 2020). Glycosides including ginsenosides further attenuate osteoclast lineage commitment by targeting NF-κB and NFATc1, key transcriptional hubs required for osteoclastogenesis (Su et al., 2022). Several flavonoids additionally reduce the RANKL/OPG ratio, thereby dampening upstream osteoclastogenic cues and limiting osteoclast specification at the initiation stage (Zhao et al., 2020). These effects collectively weaken both the initiation and the transcriptional amplification of osteoclast programs.

Beyond differentiation, PMF metabolites may also reduce osteoclast persistence. For example, glycosides from *Polygonatum sibiricum* Redouté (Asparagaceae) can promote mitochondria-dependent apoptosis via shifts in the B-cell lymphoma-2/Bcl-2-associated X (Bcl-2/Bax) balance and suppress phosphatidylinositol 3-kinase (PI3K)/serine-threonine kinase (AKT)/mTOR signaling, thereby limiting autophagy-associated survival and decreasing the resorptive capacity of mature osteoclasts (Li et al., 2024). Importantly, PMF bioactives do not merely influence osteoblasts and osteoclasts in isolation; they may also reinforce coupling signals that link resorption to formation. Certain flavonoids and polysaccharides have been reported to enhance the release/activation of matrix-sequestered factors such as TGF-β during resorption, facilitating osteoprogenitor recruitment to resorption lacunae (Shen et al., 2022). In addition, PMF metabolites can increase local IGF-1 expression, supporting osteoblast proliferation and the transition toward the formation phase (Liu et al., 2019; Rivadeneira et al., 2006). By strengthening TGF-β and IGF-1-related coupling, PMF may improve the efficiency with which resorption cavities are refilled, reducing the likelihood of “unrepaired pits” that accumulate into microarchitectural deterioration.

Notably, these osteogenic effects, particularly those involving suppression of sclerostin and activation of Wnt/β-catenin signaling, suggest a partial functional overlap with sclerostin-targeted therapies such as romosozumab, albeit with lower potency but potentially greater suitability for long-term modulation. Together, these cellular-molecular actions suggest a unifying principle: PMF substances restore BMU output through multi-node convergence, enhancing osteogenesis, suppressing osteoclastogenesis, shortening osteoclast lifespan, and improving coupling, thereby directly correcting the remodeling imbalance that defines osteoporotic bone loss.

### 6.2 Systemic hormonal crosstalk in skeletal protection

Beyond local BMU signaling, PMF substances can engage endocrine-responsive axes that shape remodeling set-points at the systemic level. A prominent route involves estrogen-mimetic phytochemicals such as puerarin and soy isoflavones, whose structural features enable selective interactions with ERs (Jagga et al., 2021; Wang et al., 2012). Functioning in a selective estrogen receptor modulators (SERMs)-like manner, these metabolites may partially counteract the postmenopausal shift toward resorption by reducing the RANKL/OPG ratio, suppressing osteoclastogenesis, and supporting estrogen-dependent anabolic signaling in bone. From a translational perspective, this multi-level suppression of the RANKL axis functionally converges with the mechanism of denosumab, although it operates through indirect and distributed regulation rather than direct ligand neutralization.

PMF may also influence estrogenic tone through microbiota-mediated metabolism. Sesame lignans (e.g., sesamin, sesamol) from *Sesamum indicum* L. (Pedaliaceae) can be converted by gut microbiota into mammalian lignans (enterodiol, enterolactone) that exhibit estrogen-like activity (Luo et al., 2022). These metabolites have been associated with improved calcium utilization and mineralization and may modulate aromatase-related pathways, potentially stabilizing endogenous estrogen availability. Consistently, sesame oil has been reported to increase circulating estrogen and aromatase levels, elevate the formation marker procollagen I C-terminal propeptide (PICP), and reduce the resorption marker N-terminal telopeptide (NTx) (Hsu et al., 2024).

In addition to ER-related routes, PMF substances may interact with vitamin K-dependent mineralization processes. Vitamin K is required for γ-carboxylation of osteocalcin (OCN), enabling high-affinity calcium binding and proper matrix mineralization (Koshihara and Hoshi, 1997). Because some natural coumarins found in PMF materials share certain structural motifs with vitamin K (Lu et al., 2022), structural convergence has been discussed as a possible basis for effects on OCN maturation/mineralization. However, given that pharmacologic disruption of vitamin K cycling (e.g., warfarin exposure) illustrates the consequences of impaired γ-carboxylation (Koshihara and Hoshi, 1997), and that limitations exist in long-term outcome interpretation (Pearson, 2007), this putative link should be framed cautiously as a mechanistic hypothesis rather than a definitive pathway.

Overall, endocrine-level interactions provide an upstream route by which PMF may reduce the systemic pro-resorptive bias characteristic of PMOP and senile OP, thereby complementing direct BMU-level actions.

### 6.3 Environmental and immune-microenvironmental modulation of remodeling

Whereas endocrine regulation largely operates through circulating hormones, environmental and immune mechanisms shape remodeling by altering the metabolic and immunological milieu in which BMU activity proceeds. PMF substances appear to engage three interconnected external nodes, the gut-bone axis, chronic low-grade inflammation, and oxidative stress, each of which can influence core remodeling hubs and shift BMU set-points.

A key route is modulation of the gut-bone axis. Ziyuglycoside II, a bioactive metabolite from *Sanguisorba officinalis* L. (Rosaceae) has been shown to attenuate bone loss in ovariectomized (OVX) mice by modulating gut microbiota and significantly increasing fecal levels of SCFAs, particularly acetic acid and propanoic acid (Zhou et al., 2024). These microbial metabolites are associated with pro-osteogenic effects, including stimulation of pre-osteoblast proliferation/differentiation and increased expression of osteogenic markers such as B-ALP, OCN, BMP-2, and Runx2. This provides *in vivo* evidence for a microbiota-dependent metabolic pathway through which PMF polysaccharides may indirectly support osteogenesis. PMF substances also mitigate chronic inflammation, a recognized driver of osteoclast activation and bone loss. Glycosides such as ginsenosides can suppress macrophage-derived cytokines (TNF-α, IL-1β, IL-6), thereby reducing inflammatory signals that amplify RANKL-driven osteoclastogenesis (Lee et al., 2023). Polysaccharides from *Polygonatum sibiricum* provide complementary immunomodulation by downregulating NF-κB signaling and alleviating inflammation-induced resorptive shifts (Wu et al., 2022). Through these anti-inflammatory actions, PMF may restore a local/systemic milieu more permissive for balanced remodeling.

A third route is reinforcement of antioxidant defenses. Excess reactive oxygen species (ROS) can impair osteoblast survival and promote osteoclast formation, making oxidative stress a central mediator of osteoporotic remodeling drift (Wang et al., 2024). PMF-derived flavonoids with phenolic hydroxyl groups serve as radical scavengers (Huzum et al., 2025; Zhang et al., 2022). For instance, robinin has been reported to inhibit RANKL-induced mitochondrial ROS and enhance antioxidant enzyme activity, thereby limiting osteoclastogenesis (Hong et al., 2021). In addition, ligustilide from *Angelica sinensis* may protect osteoblasts against oxidative stress-induced apoptosis and support differentiation via G protein-coupled receptor 30/epidermal growth factor receptor (GPR30/EGFR) signaling in an estrogen-independent manner (Yang et al., 2019).

Collectively, these gut-derived metabolic effects, anti-inflammatory actions, and antioxidant defenses remodel the external conditions under which BMU programs execute. Despite distinct upstream routes, they converge on a shared terminal mechanism, reducing RANKL-driven resorption pressure, alleviating oxidative/inflammatory suppression of osteogenesis, and improving osteocyte-mediated regulation, thereby biasing remodeling back toward structural maintenance rather than net loss. Such upstream modulation of inflammatory and metabolic drivers may further complement RANKL-targeted strategies by reducing the systemic stimuli that sustain osteoclast activation.

### 6.4 Aging-related modulation of BMSC function

Aging-related decline in BMSC number, mitochondrial integrity, and lineage fidelity is a major upstream determinant of reduced formation capacity in senile OP. PMF substances may intervene at this regenerative “source layer,” a feature that could distinguish them from conventional antiresorptives and some anabolics that primarily target mature effector cells. Myristic acid from *Myristica fragrans* Houtt. (Myristicaceae) is an illustrative example reported to delay cellular senescence and restore osteogenic potential in BMSCs by normalizing aging-associated transcriptional programs (Zhang et al., 2025). Notably, such effects have been described as context-dependent across disease settings, suggesting that PMF bioactives may modulate lineage allocation in a state-sensitive manner rather than enforcing a uniform directional push.

Mechanistically, this class of regulation may involve restoration of mitochondrial homeostasis, reduction of senescence markers, and rebalancing of osteogenic-adipogenic transcriptional programs. Flavonoids from *Eucommia ulmoides* leaves have been reported to ameliorate lipid accumulation under lipid overload by modulating SIRT-PPARγ-linked lipid metabolism signaling (Gong et al., 2022; Yan et al., 2022). Quercetin, an active metabolite of *Eucommia ulmoides* shows activity consistent with clearance of senescent BMSCs (Wang et al., 2023; Xing et al., 2022). Salvianolic acid and resveratrol are also reported to improve lipid metabolism disorders through related mechanisms, promoting osteogenic differentiation of BMSCs (Jia et al., 2022). Specifically, resveratrol has been shown to enhance mitochondrial function in senescent BMSCs via upregulation of mitofilin and improve osteogenesis in aged mice (Lv et al., 2018). By acting on BMSC aging and fate determination, PMF substances may help preserve the long-term capacity to refill resorption cavities—an essential requirement for durable remodeling balance in aging bone.

Taken together, PMF substances restore remodeling homeostasis through a systems-level mode of action: they directly rebalance BMU signaling (formation, resorption, and coupling), attenuate endocrine and immunometabolic pressures that bias remodeling toward loss, improve gut-derived and inflammatory/oxidative microenvironmental conditions, and mitigate aging-related decline in BMSC regenerative capacity. Through this multi-tier convergence, PMF bioactives can enhance osteogenesis, restrain excessive resorption, and strengthen coupling efficiency, thereby offering a mechanistically coherent basis for their anti-osteoporotic potential. This upstream preservation of osteogenic progenitor function represents a mechanistic dimension not directly addressed by current agents such as romosozumab or denosumab, which primarily target downstream effector pathways.

## 7 Clinical applications of PMF substances in OP management

Clinical studies have increasingly explored PMF substances as adjunctive, long-term options for OP management, motivated by their dietary compatibility, multi-metabolite pharmacology, and generally favorable acceptability (Table 1). In this context, it is noteworthy that several PMF interventions exert effects on pathways analogous to clinically targeted molecules such as sclerostin and RANKL, suggesting potential relevance not only as adjuncts but also as modulators of key therapeutic axes. However, the current clinical literature remains heterogeneous in design, endpoints, and overall quality; many reports are single-center and prioritize surrogate outcomes such as bone mineral density (BMD), pain scores, and bone turnover markers rather than fracture endpoints.

**TABLE 1:** Clinical applications of PMF substances in OP management.

Medication	Sample size (n)	Duration	Control drug	Primary endpoint	Risk of bias	References
*Epimedium brevicornu*	85	24 months	Placebo	BMD	Low	Zhang et al. (2007)
	58	6 weeks	Placebo	Safety, pharmacokinetics	Low	Yong et al. (2021)
	120	6 months	Calcium carbonate, vitamin D, and zoledronic acid	BMD, bone metabolism markers, periodontal health indicators, inflammatory markers, scale scores	Moderate (retrospective)	Guo et al. (2024)
Soy isoflavone	78	12 months	Placebo	BMD	Low	Lambert et al. (2017)
	106	3 months	Placebo	Plasma lipids, urinary indices of bone resorption	Moderate (high dropout rate)	Dalais et al. (2003)
*Prunus domestica* L. (Rosaceae)	35	3 months	Placebo	Trabecular bone score (TBS), BMD	Low	George et al. (2022)
*Cornus mas*	84	8 weeks	Placebo	Biomarkers of inflammation and bone metabolism	Low	Aryaeian et al. (2021)
Garlic	44	1 month	Placebo	Pro-inflammatory cytokines	Moderate (small sample/short duration)	Mozaffari-Khosravi et al. (2012)
Formula Xian Ling Gu Bao	180	12 months	Placebo	BMD, safety	Low	Zhu et al. (2012)
Bo-gu Ling	150	12 months	Placebo	BMD	Low	Leung et al. (2011)
Kidney-tonifying herbal Fufang	194	5 years	Placebo	BMD, fracture	Low	Deng et al. (2012)
Liuwei Dihuang pill	205	6 months	Healthy control	CLCF1 expression, JAK/STAT pathway markers	Low	Ge et al. (2018)
Kudzu Flower–Mandarin peel	84	12 weeks	Placebo	Biomarkers of bone metabolism, safety	Low	Kim et al. (2020)
Shugan Jiangu recipe	38	6 months	Caltrate D	BMD, biomarkers of bone metabolism	Moderate (small sample size)	Li et al. (2014)
Jianpi Bushen formula	130	12 months	Caltrate D	BMD, biomarkers of bone metabolism	Low	Dai et al. (2018)

In the clinical application of single-herb PMF preparations for OP, *Epimedium brevicornu* is one of the most extensively investigated herbs. A 24-month randomized, double-blind, placebo-controlled trial demonstrated that a daily phytoestrogenic flavonoid regimen derived from *Epimedium brevicornu* significantly preserved lumbar spine and femoral neck BMD in postmenopausal women without inducing endometrial hyperplasia (Zhang et al., 2007). When *Epimedium brevicornu* flavonoids were added to conventional therapy (calcium, vitamin D and zoledronic acid), they not only further improved BMD at the lumbar spine and proximal femur but also ameliorated periodontal indicators and suppressed bone resorption markers such as β-CTx (Guo et al., 2024). Mechanistically, a clinical pharmacokinetic study confirmed that *Epimedium brevicornu* prenylated flavonoids favorably modulate bone turnover by regulating the osteoclast adaptor protein TRAF6 and bone-specific alkaline phosphatase (Yong et al., 2021). Soy isoflavones, another well-characterized phytoestrogen source, exert bone-sparing effects that can be enhanced by modulating the gut microbiota: combining red clover extract with probiotics improved bone status in osteopenic postmenopausal women by increasing isoflavone bioavailability, inhibiting bone resorption and improving estrogen metabolism (Lambert et al., 2017). Although short-term soy protein supplementation only modestly influenced BMD, it reduced urinary deoxypyridinoline and favorably affected lipid profiles and bone turnover balance (Dalais et al., 2003). Beyond phytoestrogen-rich plants, several other PMF items act primarily through anti-inflammatory and antioxidant pathways. *Cornus mas* L. (Cornaceae) extract significantly decreased TNF-α and improved OCN in PMOP (Aryaeian et al., 2021). Daily intake of dried plums (100 g for 3 months) markedly inhibited bone resorption and improved bone strength markers in men, extending the protective potential to male age-related bone loss (George et al., 2022). Garlic *Allium sativum* L. (Amaryllidaceae) tablets reduced the pro-inflammatory cytokine IL-6 in PMOP, underscoring the role of systemic inflammation control in OP prevention (Mozaffari-Khosravi et al., 2012). Together, these studies provide clinical evidence that selected PMF agents—through phytoestrogenic, immunomodulatory, and gut-bone-axis mechanisms—can serve as effective adjuncts or preventive strategies in OP management.

In parallel with single agents, clinical evidence supports the use of Chinese herbal formulas (Fufang) founded on the principle that “the kidney governs bone” and on syndrome differentiation (Zheng). A multicenter randomized controlled trial (RCT) demonstrated that the proprietary formula Xian Ling Gu Bao (containing *Epimedium brevicornu, Dipsacus* L. (Caprifoliaceae), *Salvia miltiorrhiza* Bunge (Lamiaceae), and *Anemarrhena asphodeloides* Bunge (Asparagaceae)) significantly increased lumbar spine BMD at 6 months and proved safe during 1 year of daily administration in PMOP (Zhu et al., 2012). An innovative three-herb kidney-tonifying formula, Bo-gu Ling (containing *Epimedium brevicornu, Ligustrum lucidum* W.T.Aiton (Oleaceae), and *Psoralea corylifolia* L. (Fabaceae), effectively improved lumbar spine and hip BMD over 12 months in women who had been postmenopausal for more than 10 years, with particular benefit in osteopenic patients (Leung et al., 2011). Crucially, a 5-year follow-up study revealed that sustained use of a kidney-tonifying herbal formula (containing *Epimedium brevicornu, Rehmannia glutinosa, Dioscorea polystachya* Turcz. (Dioscoreaceae), *Cornus officinalis* Siebold & Zucc. (Cornaceae), *Drynaria fortunei* (Kunze ex Mett.) J.Sm. (Polypodiaceae), and *Morinda officinalis* F.C.How (Rubiaceae)) markedly reduced the rate of bone loss and the incidence of fragility fractures in PMOP (Deng et al., 2012), providing rare long-term evidence. Reflecting individualized syndrome differentiation, Liuwei Dihuang Pill (containing *Rehmannia glutinosa, Cornus officinalis*, and *Dioscorea polystachya*), indicated for Kidney-Yin deficiency, not only improved clinical outcomes but was also shown to upregulate cardiotrophin-like cytokine factor 1 (CLCF1) expression via the Janus kinase/signal transducer and activator of transcription (JAK/STAT) pathway, thereby inhibiting bone resorption and promoting bone formation (Ge et al., 2018). For the special population of breast cancer patients with aromatase inhibitor-associated bone loss (AIBL), the addition of the Shugan Jiangu Recipe (containing *Bupleurum chinense* DC. (Apiaceae) *Paeonia* × *suffruticosa* Andrews (Paeoniaceae), and *Dipsacus asper* Wall. ex DC. (Caprifoliaceae) to calcium and vitamin D significantly elevated lumbar spine BMD and reduced the bone turnover marker C-terminal telopeptide of type II collagen (CTX-II) after 6 months (Li et al., 2014), and the bone-protective effect of the Jianpi Bushen Formula (containing *Astragalus membranaceus, Dioscorea polystachya, Atractylodes macrocephala* Koidz. (Asteraceae), and *Eucommia ulmoides*) was further confirmed in postmenopausal breast cancer survivors (Dai et al., 2018). Extending into herb-food combinations, a blend of *Pueraria lobata* (rich in isoflavones) and *Citrus reticulata* Blanco (Rutaceae) extract alleviated climacteric symptoms and concurrently improved bone turnover markers in perimenopausal women (Kim et al., 2020). Collectively, these studies demonstrate that syndrome-differentiation-based Chinese herbal formulas—whether classical, innovative, or adapted for conditions such as AIBL—can provide sustained, multi-targeted clinical benefits in osteoporosis management.

Safety remains central to long-term OP strategies. The relatively moderate and multi-target nature of PMF metabolites, compared with highly specific biologics targeting sclerostin or RANKL, may contribute to a lower risk of severe adverse events and rebound phenomena, supporting their potential role in long-term management. However, clinically meaningful risks can emerge with chronic high intake or inappropriate use. Glycyrrhizin may cause pseudoaldosteronism under prolonged high exposure (Ceccuzzi et al., 2023; Chi et al., 2023; Zhai et al., 2025), and coumarins derived from *Cinnamomum cassia* have been linked to dose-dependent hepatotoxicity (Iwata et al., 2016). Regulatory agencies have established a tolerable daily intake (TDI) of approximately 0.1 mg/kg body weight. Importantly, the doses employed in several preclinical studies investigating PMF metabolites often exceed levels achievable through typical dietary intake or traditional use, which may limit the direct translational relevance of the observed toxic effects. For instance, high-dose exposure in experimental models may not accurately reflect real-world consumption patterns of coumarin-containing PMFs such as *Cinnamomum cassia*. Therefore, while coumarin-related hepatotoxicity warrants careful consideration, the actual clinical risk is highly context-dependent and influenced by dose, duration of exposure, and formulation characteristics. In addition, exogenous contamination, processing variability, and potential herb-drug interactions underscore the need for rigorous quality control and risk monitoring. Accordingly, contemporary analytical and pharmacokinetic approaches, including ultra-high performance liquid chromatography-tandem mass spectrometry (UPLC-MS/MS), metabolomics, and interaction prediction models, are increasingly applied to standardize phytochemical profiles, detect contaminants, and optimize dosing safety (Dong et al., 2015; Ezrari et al., 2025; Wang et al., 2021; Zhou et al., 2015). Looking forward, high-quality evidence will require multicenter, adequately powered RCTs with harmonized formulations and endpoints, longer follow-up, and explicit evaluation of add-on strategies combining PMF with antiresorptive or anabolic agents to improve sustained benefit while maintaining safety.

Given that patients with OP are typically maintained on long-term pharmacotherapy—including bisphosphonates, calcium supplements, and vitamin D—co-administration with PMF substances introduces a clinically relevant but insufficiently characterized layer of interaction. These interactions may arise at multiple levels. At the pharmacokinetic level, certain PMF-derived metabolites, particularly polyphenols and phytates, can chelate divalent cations and reduce the intestinal absorption of calcium supplements, thereby attenuating their contribution to bone mineralization (Schlemmer et al., 2009). In addition, the already limited oral bioavailability of bisphosphonates may be further compromised by herbal products that alter gastrointestinal pH or transporter activity (Dissette et al., 2010). At the metabolic level, PMF constituents may modulate cytochrome P450 enzymes or gut microbiota-mediated biotransformation, potentially affecting drug exposure (Doostdar et al., 2000). At the pharmacodynamic level, interactions may involve convergence on shared biological pathways. For example, coumarin-containing materials (e.g., *Cinnamomum cassia*) may interfere with vitamin K-dependent processes or potentiate anticoagulant effects, while phytoestrogenic compounds such as isoflavones and puerarin may exert additive or modulatory effects on ER signaling, potentially influencing the efficacy or safety profile of hormone-related or antiresorptive therapies. Collectively, these observations underscore the need for systematic evaluation of herb–drug interactions in OP management. From a translational perspective, careful medication reconciliation, appropriate dose spacing, and monitoring of biochemical markers are warranted in clinical practice. Future studies should integrate pharmacokinetic and systems pharmacology approaches to better define interaction networks and support evidence-based co-administration strategies.

## 8 Conclusion

PMF substances offer a uniquely integrative strategy for OP management, acting across multiple regulatory layers, including cellular signaling, endocrine modulation, immunometabolic balance, and aging-related stem-cell dynamics, to restore the fidelity of bone remodeling. Their bioactive metabolites consistently promote osteogenesis, suppress excessive resorption, and reshape the systemic microenvironment that governs BMU behavior, providing a systems-level therapeutic profile distinct from conventional single-target agents. Coupled with their generally favorable safety profile and suitability for long-term use, PMF materials represent a compelling adjunct and potential standalone option in chronic OP care.

Despite the substantial body of mechanistic insights derived from preclinical studies, it is important to recognize that a large proportion of the current evidence base for PMF bioactives relies on *in vitro* systems and OVX rodent models. While the OVX model is widely accepted as a classical model of PMOP due to its ability to mimic estrogen deficiency, several critical translational limitations must be acknowledged when extrapolating these findings to human disease. First, OVX models incompletely recapitulate the skeletal phenotype of human PMOP, particularly in terms of compartment-specific bone loss. In rodents, bone loss following OVX occurs rapidly and predominantly affects trabecular bone, whereas in humans, postmenopausal bone loss involves a more complex and progressive pattern, including significant cortical thinning, increased cortical porosity, and impaired bone quality (Kalu, 1991; Plank et al., 2025). This discrepancy may lead to an overestimation of therapeutic efficacy, especially for interventions primarily targeting trabecular remodeling. Second, the temporal dynamics of bone loss differ markedly between OVX models and human disease. OVX-induced bone loss occurs over weeks, representing an acute, high-turnover state, whereas human PMOP develops over years to decades under the influence of aging, cumulative microdamage, and long-term endocrine and metabolic changes. Consequently, short-term preclinical studies may fail to capture the chronic remodeling imbalance and long-term treatment responses relevant to clinical settings. Third, the hormonal and systemic environment in OVX animals is oversimplified compared with postmenopausal women. Human PMOP is shaped not only by estrogen deficiency but also by complex interactions involving aging-related changes in androgen levels, vitamin D metabolism, parathyroid hormone regulation, immune aging (inflammaging), and comorbidities. In contrast, OVX models isolate estrogen withdrawal as the primary driver, thereby neglecting the multifactorial nature of bone remodeling dysregulation in humans. Fourth, species-specific differences in bone biology further limit translational relevance. Rodents exhibit higher bone turnover rates, distinct skeletal microarchitecture, and differences in osteocyte network organization and remodeling patterns compared with humans. These intrinsic biological differences may influence both disease progression and pharmacological responsiveness. Beyond limitations of the OVX model itself, the current preclinical literature on PMF bioactives is also constrained by heterogeneity in experimental design, including variability in extraction methods, dosing regimens, treatment duration, and outcome measures. Moreover, many studies rely heavily on surrogate endpoints such as BMD or biochemical markers, with limited assessment of bone quality, microarchitecture, and mechanical strength, which are more directly linked to fracture risk.

Future research should therefore prioritize improving the translational relevance of preclinical models and strengthening the clinical evidence base. Several key directions are warranted. First, the integration of more representative models, including aged animals, glucocorticoid-induced osteoporosis models, and models incorporating metabolic or inflammatory comorbidities, may better capture the multifactorial nature of human OP. Second, longer-term studies assessing both trabecular and cortical compartments, as well as bone quality parameters (e.g., microarchitecture, porosity, biomechanical properties), are needed to more accurately predict clinical outcomes. Third, mechanistic studies should increasingly adopt multi-omics and systems biology approaches to delineate how PMF bioactives interact with complex regulatory networks, including the gut-bone axis, immune system, and aging-related pathways. Fourth, standardization of PMF preparations, including chemical characterization, bioavailability assessment, and dose optimization, is essential to enhance reproducibility and facilitate clinical translation. Most importantly, well-designed, multicenter RCTs with sufficient sample size, long-term follow-up, and clinically meaningful endpoints (e.g., fracture incidence) are urgently needed. Future clinical studies should also explore PMF as adjunctive therapies in combination with established antiresorptive or anabolic agents, with a focus on long-term safety, adherence, and real-world effectiveness. With these developments, PMF-based interventions have the potential to evolve into scientifically validated, internationally applicable strategies for the long-term prevention and treatment of OP.
