# Supplementary material for: Plants used in medicines and foods in osteoporosis: mechanistic insights into bone-remodeling homeostasis and clinical evidence
Source: Front Pharmacol. 2026 Jun 1;17:1839782. doi: 10.3389/fphar.2026.1839782 (PMC13265558; doi:10.3389/fphar.2026.1839782)
Supplement: Supplementary file 1 [file Table1.docx]

**Supplemental Table 1.** Action mechanisms of MFH in OP treatment.

| Regulatory Target | Mechanism | MFH Substance | Bioactive | Key Pathway |
| --- | --- | --- | --- | --- |
| Cellular-molecular regulation | Osteogenesis | Pueraria lobata | Puerarin | Wnt/β-catenin↑→β-catenin nuclear translocation↑→Runx2/Osx↑ |
|  | Osteogenesis | Glycine max | Soy isoflavones | Wnt/β-catenin↑→β-catenin nuclear translocation↑→Runx2/Osx↑ |
|  | Osteogenesis | Epimedium brevicornum | Icariin | Wnt/β-catenin↑→β-catenin nuclear translocation↑→Runx2/Osx↑ |
|  | Osteogenesis | Rehmannia glutinosa | Extracts | Wnt/β-catenin↑→β-catenin nuclear translocation↑→Runx2/Osx↑ |
|  | Osteogenesis | Lycium barbarum | LBP1C-2 | BMP-2/SMAD↑ |
|  | Osteogenesis | Eucommia ulmoides | EuOCP3 | BMP-2/SMAD↑ |
|  | Osteogenesis | Cinnamomum cassia | (E)-cinnamaldehyde | BMP-2↑, Runx2↑ |
|  | Anti-osteoclastogenesis | Angelica sinensis | Coumarin-containing extracts | RANKL→NFATc1↓→CTSK/TRAP↓ |
|  | Anti-osteoclastogenesis | Crataegus pinnatifida | Coumarin-containing extracts | RANKL→NFATc1↓→CTSK/TRAP↓ |
|  | Anti-osteoclastogenesis | Panax ginseng | Ginsenosides | NF-κB↓, NFATc1↓ |

**Supplemental Table 1** (continued)

| Regulatory Target | Mechanism | MFH Substance | Bioactive | Key Pathway |
| --- | --- | --- | --- | --- |
|  | Reduced osteoclast survival | Polygonatum sibiricum | Glycosides | Bcl-2/Bax-mediated apoptosis↑; PI3K/AKT/mTOR↓ |
|  | Enhanced coupling | Others | Certain flavonoids/polysaccharides | TGF-β↑, IGF-1↑ |
| Systemic hormonal crosstalk | Estrogen-like (natural SERM-like) | Pueraria lobata | Puerarin | Selective ER activation; RANKL/OPG↓ |
|  | Estrogen-like (natural SERM-like) | Glycine max | Soy isoflavones | ER-related signaling |
|  | Estrogen-like (gut microbiota-amplified) | Sesamum indicum | Sesamin/sesamol →enterodiol/enterolactone | Microbial metabolism; aromatase modulation |
|  | Estrogen/aromatase modulation | Sesamum indicum | Sesame oil | PICP↑, NTx↓ |
|  | Vitamin K-related mineralization | Others | Coumarins | OCN γ-carboxylation (mechanistic basis) |
| Environmental and immune-microenvironmental modulation | Gut-bone axis | Lycium barbarum | Polysaccharides | SCFAs↑ |

**Supplemental Table 1** (continued)

| Regulatory Target | Mechanism | MFH Substance | Bioactive | Key Pathway |
| --- | --- | --- | --- | --- |
|  | Anti-inflammatory | Panax ginseng | Ginsenosides | TNF-α, IL-1β, IL-6↓ |
|  | Anti-inflammatory | Polygonatum sibiricum | Polysaccharides | NF-κB↓ |
|  | Antioxidant (anti-osteoclast) | Robinia pseudoacacia | Robinin | Mitochondrial ROS↓ |
|  | Antioxidant/ osteoblast protection | Angelica sinensis | Ligustilide | GPR30/EGFR signaling |
|  | Antioxidant | Others | Certain flavonoids with multiple phenolic hydroxyl groups | ROS scavenging |
| Aging-related modulation | Anti-senescence and osteogenic regulation | Myristica fragrans | Myristic acid | BMSC senescence programs |
|  | Lipid metabolism correction | Eucommia ulmoides | Flavonoids | SIRT-PPARγ axis |
|  | Senolytic tendency | Eucommia ulmoides | Quercetin | Clearance of senescent BMSCs |
|  | Lipid metabolism-mediated osteogenesis | Salvia miltiorrhiza | Salvianolic acid | Lipid metabolic regulation |

**Supplemental Table 1** (continued)

| Regulatory Target | Mechanism | MFH Substance | Bioactive | Key Pathway |
| --- | --- | --- | --- | --- |
|  | Mitochondrial functional improvement | Mori Fructus | Resveratrol | Mitofilin↑ |
