# Supplementary material for: Plants used in medicines and foods in osteoporosis: mechanistic insights into bone-remodeling homeostasis and clinical evidence
Source: Front Pharmacol. 2026 Jun 1;17:1839782. doi: 10.3389/fphar.2026.1839782 (PMC13265558; doi:10.3389/fphar.2026.1839782)
Supplement: Supplementary file 2 [file Table2.docx]

**Supplemental Table 2. Summary of experimental design parameters of preclinical OP studies on PMF cited in this review**

| PMFs | Type of extract | Dose range tested | Active concentration | Model | Control | Duration |
| --- | --- | --- | --- | --- | --- | --- |
| Epimedium brevicornu Maxim. | Icariin | - | - | BMSC (vitro) | - | - |
|  |  | 1, 3, 5 mg/kg | 5 mg/kg | OPG−/− mice (vivo) | WT+Veh;  KO+Veh | 5 days |
| Rehmannia glutinosa (Gaertn.) Libosch. ex DC. | Extract | 2.5 g/1 mL/100 g | 2.5 g/1 mL/100 g | OVX rat (vivo) | Sham+Veh;  OVX+Veh; OVX+estradiol valerate | 14 weeks |
| Lycium barbarum Lam. | LBP1C-2 | - | - | MC3T3-E1 (vitro) | - | - |
|  |  | 40 mg/kg | 40 mg/kg | Aging mice (vivo) | Adult+Veh; Aging+Veh; Adult+ LBP1C-2 | 4 months |
|  |  | 40 mg/kg | 40 mg/kg | OVX mice (vivo) | Sham+Veh;  OVX+Veh | 4 months |
| Eucommia ulmoides Oliv. | EuOCP3 | - | - | MC3T3-E1 (vitro) | - | - |
|  |  | 100, 300mg/kg | 100, and 300mg/kg | Dex mice (vivo) | Veh; EuOCP3; Dex+Veh; Dex+E2; Dex+ EuOCP3 | 49 days |
|  | Flavonoids | - | - | HepG2 (vitro) | - | - |
|  | Extract | 250, 1000 mg/kg | 250 mg/kg | Broiler (vivo) | Basal diet | 51 days |
|  | Quercetin | - | - | BMSC (vitro) | - | - |
|  |  | 5, 50 mg/kg | 5, 50 mg/kg | Aging rat (vivo) | Young; Middle-aged | 4 months |
|  |  | 5, 50 mg/kg | 5, 50 mg/kg | OVX rat (vivo) | Sham+Veh;  OVX+Veh | 4 months |
| Cinnamomum cassia (L.) J.Presl | Extract | - | - | MC3T3-E1 (vitro) | - | - |
|  |  | 25 g/kg | 25 g/kg | OVX mice (vivo) | Sham;  OVX | 12 weeks |
| Lotus lalambensis Schweinf. | Coumarin derivative | - | - | BMSC (vitro) | - | - |
| Angelica sinensis (Oliv.) Diels | Extract | - | - | BMM (vitro) | - | - |
|  | Angelicin | - | - | RAW264.7 (vivo) | - | - |
|  |  | 5, 10, 20 mg/kg | 5, 10, 20 mg/kg | OVX rat (vivo) | Sham+Veh; OVX+alendronate | 3 months |
|  | Ligustilide | - | - | MC3T3-E1; BMSC (vitro) | - | - |
|  |  | 10 μM | 10 μM | Prednisolone zebrafish (vivo) | Veh; Ligustilide; Pred;  Pred+E2 | 6 days |
| Angelica archangelica L. | Imperatorin | - | - | BMSC (vivo) | - | - |
|  |  | 20 mg/kg/ | 20 mg/kg/ | OVX rat (vivo) | Sham+Veh; OVX+Veh | 12 weeks |
| Scutellaria baicalensis Georgi | Baicalin | 0.22, 11 μM/L | 0.22, 11 μM/L | Dex zebrafish (vivo) | Veh; Dex; Dex+etidronate disodium; Baicalin | 60 days |
| Aster tataricus L.f. | Aster saponin A2 | - | - | RAW264.7; BMM (vivo) | - | - |
| Polygonatum sibiricum Redouté | Dioscin | - | - | EC; HEK293 (vivo) | - | - |
| Glycine max (L.) Merr. | Isoflavone | - | - | MC3T3-E1 (vitro) | - | - |
| Pueraria lobata (Willd.) Ohwi | Puerarin | 20 mg/kg | 20 mg/kg | OVX rat (vivo) | Sham+Veh; OVX+Veh | 12 weeks |
| Sesamum indicum L. | Sesame oil | 0.25, 0.5 mL/kg | 0.25, 0.5 mL/kg | OVX rat (vivo) | Sham; OVX | 4 months |
| Sanguisorba officinalis L. | Ziyuglycoside II | 7.5, 15, 30 mg/kg | 7.5, 15, 30 mg/kg | OVX mice (vivo) | Sham+Veh; OVX+Veh | 6 weeks |
| Polygonatum sibiricum Redouté | Polysaccharide | - | - | Chondrocytes (vitro) | - | - |
| Vinca erecta Regel & Schmalh. | Robinin | - | - | BMM (vitro) | - | - |
|  |  | 6 mg/kg | 6 mg/kg | OVX mice (vivo) | Sham+Veh; OVX+Veh | 6 weeks |
| Myristica fragrans Houtt. | Myristic acid | - | - | BMSC; LFC (vitro) | - | - |
| Veratrum nigrum L. | Resveratrol | - | - | BMSC (vitro) | - | - |
